# Retrospective Analysis of the Outcomes of Mycophenolate Therapy in Retarding the Progression of Immunoglobulin A Nephropathy (REMISSION IgA)

**DOI:** 10.25259/IJN_502_2024

**Published:** 2025-02-25

**Authors:** Chilaka Rajesh, Selvin Sundar Raj Mani, Utkarash Mishra, Sanjeet Roy, N Jansi Rani, Ankit Jain, T Jayaprakash, B Dhivakar, Manish Lalwani, Nisha Jose, Joseph Johnny, Jeethu Joseph Eapen, Athul Thomas, Elenjickal Elias John, Vinoi George David, Santosh Varughese, Suceena Alexander

**Affiliations:** 1 Departments of Nephrology, Christian Medical College, Vellore, Tamil Nadu, India; 2 Departments of Pathology, Christian Medical College, Vellore, Tamil Nadu, India; 3 Departments of Biostatistics, Christian Medical College, Vellore, Tamil Nadu, India

**Keywords:** Angiotensin receptor blockers, End stage kidney disease, IgA Nephropathy, Mycophenolate therapy, Steroids

## Abstract

**Background:**

IgA nephropathy (IgAN) is the most common form of glomerulonephritis globally, and a leading cause of end-stage kidney disease (ESKD). In India, IgAN accounts for 10-15% of kidney biopsies, often with nephrotic syndrome and renal impairment. Steroids are the mainstay of treatment, though the role of mycophenolate (MPA) is less explored. This study investigated the outcomes of MPA in IgAN.

**Materials and Methods:**

This retrospective study included patients ≥18 years old with biopsy-proven IgAN from January 1, 2010, to December 31, 2017, and eGFR >15 mL/min/1.73m^2^. Patients were treated with angiotensin receptor blockers (ARBs), steroids, and MPA based on disease activity. The cohort was divided into three groups: ARB, ARB+prednisolone, and ARB+prednisolone+MPA. eGFR and proteinuria data were collected at baseline, 12, 36, and 60 months and compared between groups.

**Results:**

IgAN comprised 16.7% of kidney biopsies. The mean age of patients was 36.6±10.1 years, with 88.9% having hypertension. Over 60 months, eGFR decreased by 2 mL/min/1.73m^2^ in both groups, with 16.4% of patients in the MPA group progressing to ESKD, compared to 18.4% in the steroid group (p=0.42).

**Conclusion:**

MPA, in addition to steroids and ARBs, may help mitigate IgAN progression, though there were no differences in ESKD progression. Further randomized controlled trials are needed to validate the role of MPA in IgAN treatment.

## Introduction

IgA nephropathy (IgAN) is the most prevalent form of glomerulonephritis and a leading cause of end-stage kidney disease (ESKD) globally. In India, IgAN accounts for 10% to 15% of all kidney biopsies, often presenting with a high incidence of nephrotic syndrome and renal impairment.^1–6^ The Glomerular Research and Clinical Experiments–IgA Nephropathy in Indians (GRACE-IgANI) study, conducted at our center, established the first prospective South Asian cohort on IgAN. This study reported the initial clinical, biochemical, and histopathological characteristics of GRACE-IgANI, along with the baseline risk assessment for disease progression.^7^ The present investigation aims to evaluate the efficacy of mycophenolate mofetil therapy in mitigating the progression of IgA nephropathy.

## Materials and Methods

**Figure 1:**
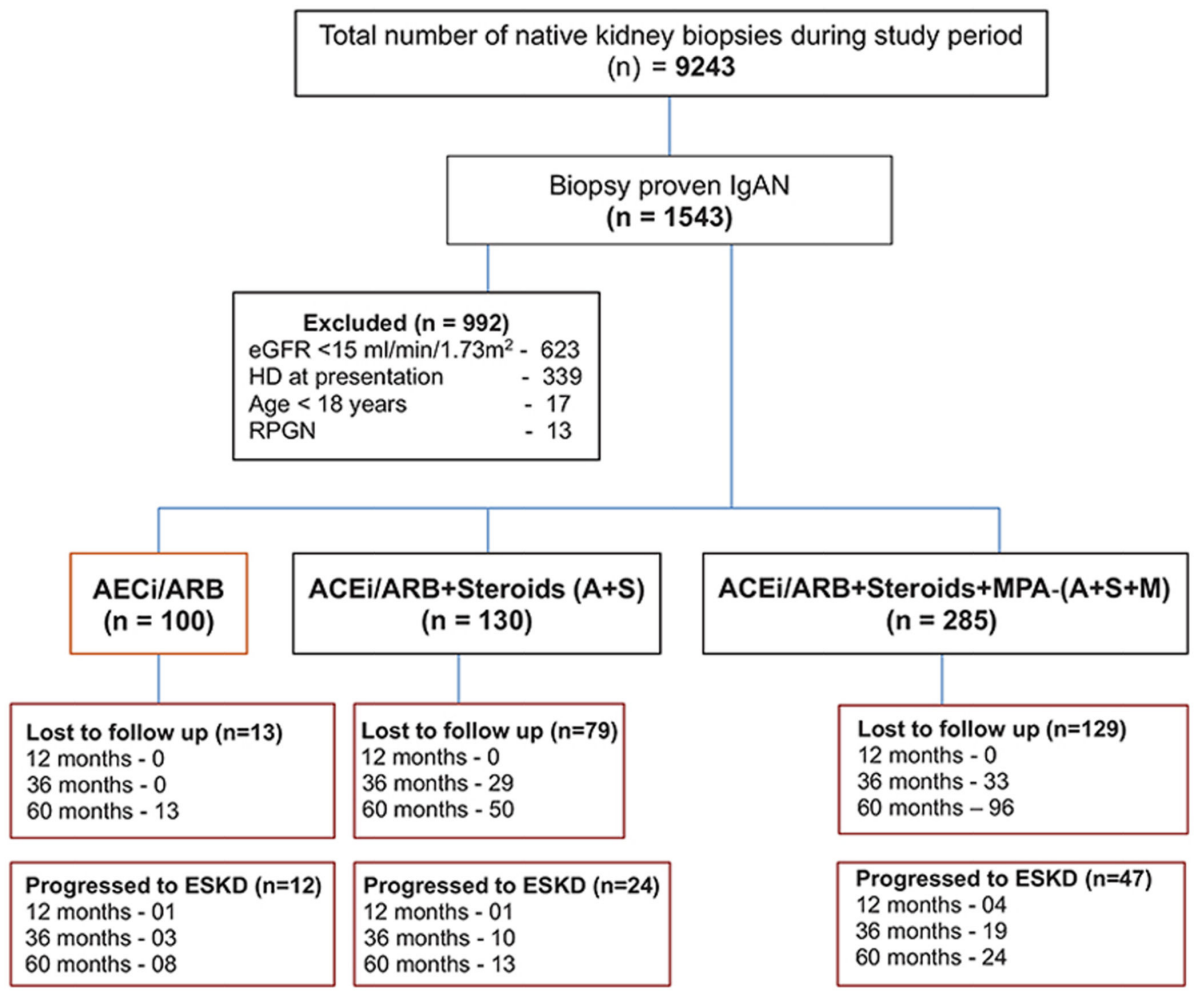
Study flow chart. ACEi: Angiotensin converting enzyme inhibitors, ARB: Angiotension receptor blockers, eGFR: Estimated glomerular filtration rate, ESKD: End stage kidney disease, HD: Hemodilaysis, IgAN: Immunoglobulin A neprhopathy, MMF: Mycophenolic acid, RPGN: Rapidly progressive glomerulonephriti.

This retrospective, single-center study was conducted at Christian Medical College, Vellore, Tamil Nadu, India after approval from the Institutional Review Board. Since it is a retrospective study, patient consent was not required. The study included adult patients (18 years and older) with biopsy-confirmed IgA nephropathy who were observed between January 1, 2010, and December 31, 2017, and had an estimated glomerular filtration rate (eGFR) greater than 15 mL/min/1.73m^2^. Data were retrieved from the electronic medical records within the Departments of Nephrology and Nephropathology. Figure 1 illustrates the study’s flowchart. Treatment protocols included angiotensin receptor blockers (ARBs)/angiotensin-converting enzyme inhibitors (ACEi), oral steroids, and mycophenolic acid (MPA), tailored according to the treating physician’s decision based on the patient’s eGFR and biopsy findings. Patients were categorized into three groups based on the treatment received: ARB group, ARB & steroid group, and ARB, steroid & MPA group. Maximum tolerated doses of ARB/ACEi were administered in all groups before initiating immunosuppression treatment. Steroid therapy was initiated at 1 mg/kg body weight for two months, followed by gradual tapering over four months in the steroid group. Two treatment protocols were followed for the initiation of mycophenolate therapy. In the first protocol, patients received both steroid and MPA together (prednisolone at 0.5 mg/kg and mycophenolate at a dosage of 500 mg to 1 gram twice daily [equivalent dose of sodium preparation]), with steroids tapered over three months and MPA continued for 3–5 years. In the second protocol, patients received mycophenolate at the end of two months during the tapering. The dose of mycophenolate was adjusted according to the MPA area under the curve (AUC) level, with a target of 30–60 mg·h/L, as adopted from kidney transplant protocols. MPA levels were checked using high-performance liquid chromatography (HPLC). Sodium-glucose cotransporter inhibitors (SGLT2i) were not administered to any patients, as they were not part of the standard of care during the study period. Data on eGFR and urine protein-creatinine ratio (Up/Uc) were collected at diagnosis, 12, 36, and 60 months across the three treatment groups. The data were collected using EpiData version 4.6, and statistical analysis was performed using STATA version 16. Categorical variables were presented as frequencies and proportions, and continuous variables were expressed as means with standard deviations or medians with interquartile ranges. Comparative analyses were performed for the steroid and MPA groups, as the ARB group had higher baseline eGFR with minimal interstitial fibrosis and tubular atrophy (IFTA) on the biopsy. The comparative analysis for the steroid and MPA groups was conducted using the Mann-Whitney U test for continuous variables and the Chi-squared test for categorical variables. Kaplan-Meier survival analysis was performed for patients who had at least 12 months of follow-up.

## Results

During the study period, 9,243 patients underwent native kidney biopsies, of whom 1,543 (16.7%) were diagnosed with IgA nephropathy. A total of 992 (64%) patients were excluded from the study, with 623 patients (62.8%) exhibiting an eGFR < 15 mL/min/1.73m^2^ and 339 patients (34.1%) having ESKD requiring dialysis at presentation. Patients <18 years of age accounted for 17 (1.7%), while 13 (1.31%) presented with rapidly progressive glomerulonephritis (RPGN). The remaining 543 patients included in the study were categorized based on treatment into three groups: ARB group (100 patients), ARB and steroid (A+S) group (130 patients), and ARB, steroid and mycophenolate mofetil (MMF) group (285 patients). Hypertension was present at presentation in 88.9% (458/515) of patients across all three groups. Other clinical features included non-visible hematuria in 41% (212/515), hematuria with proteinuria and a rise in serum creatinine in 39% (201/515), and nephrotic-range proteinuria in 20% (102/515).

### Baseline characteristics

The baseline characteristics of the three groups are summarized as follows and detailed in Table 1.

In the ARB group (n = 100), the mean age was 34.2 ± 10.3 years, with a male-to-female ratio of 1.5:1. A history of type 2 diabetes was present in 5% of patients. The median baseline eGFR was 80.21 mL/min/1.73m^2^ (IQ: 50.98–106.09), and the median Up/Uc ratio was 0.92 (IQ: 0.47–1.58). Kidney biopsy findings revealed mesangial hypercellularity in 17% and C3 co-deposition in 63% of patients. Interstitial fibrosis and tubular atrophy (IFTA) was categorized as mild in 72% of patients, moderate in 20%, and severe in 8%.

In the A+S group (n = 130), the mean age was slightly higher at 37.01 ± 10.37 years, with a male-to-female ratio of 2.3:1. A history of type 2 diabetes was noted in 5.3% of patients. The baseline median eGFR was 48.73 mL/min/1.73m^2^ (IQ: 35.77–75.84), and the median Up/Uc ratio was 1.59 (IQ: 0.79–2.54). Kidney biopsy findings showed mesangial hypercellularity in 60% of patients, focal endocapillary proliferation in 26.1%, and diffuse endocapillary proliferation in 13.8%. C3 co-deposition was observed in 66.1% of patients. IFTA severity was mild in 17.6%, moderate in 73%, and severe in 9.2% of patients.

In the A+S+M group (n = 285), the mean age was 37.42 ± 9.94 years, with a male-to-female ratio of 2.03:1. A history of type 2 diabetes was observed in 3.5% of patients. The baseline median eGFR was 45.60 mL/min/1.73m^2^ (IQ: 32.00–62.85), and the median Up/Uc ratio was 1.88 (IQ: 0.97–3.29). Kidney biopsy findings revealed mesangial hypercellularity in 49.8% of patients, focal endocapillary proliferation in 42.4%, and diffuse endocapillary proliferation in 7.7%. C3 co-deposition was present in 67.3% of patients. IFTA was categorized as mild in 27.3% of patients, moderate in 35%, and severe in 37.5%.

### Course and Outcomes

In the ARB group, the median eGFR showed stability over the follow-up period, with values of 85.09 mL/min/1.73m^2^ at both 12 and 36 months, and 81.90 mL/min/1.73m^2^ at 60 months. The Up/Uc ratio improved progressively, declining from 0.77 at 12 months to 0.56 at 36 months and 0.35 at 60 months. In terms of treatment, 32% of patients received ACE inhibitors (ACEi), of whom 7 developed ACEi-induced dry cough that required drug discontinuation, while 68% were on ARBs. Severe hyperkalemia occurred in 18 patients, necessitating treatment discontinuation. By the end of 60 months, 13 patients were lost to follow-up, and 12 patients (9 males and 3 females) progressed to end-stage kidney disease (ESKD).

In the A+S group, the median eGFR initially improved to 57.74 mL/min/1.73m^2^ at 12 months but declined to 50.00 mL/min/1.73m^2^ at 36 months and 49.21 mL/min/1.73m^2^ at 60 months. The Up/Uc ratio showed an initial decrease to 0.38 at 12 months but worsened slightly to 0.48 at 36 months and 0.67 at 60 months. Steroid-related complications included steroid-induced diabetes in 18 patients (13.8%) and avascular necrosis (AVN) of the hip in three patients. By the end of 60 months, 31 patients (23.8%) were lost to follow-up, and 24 (16 males and 8 females, 18.4%) progressed to ESKD.

In the A+S+M group, the median eGFR remained stable at 48.71 mL/min/1.73m^2^ at 12 months and 48.90 mL/ min/1.73m^2^ at 36 months but declined to 43.60 mL/ min/1.73m^2^ at 60 months. The Up/Uc ratio showed improvement, with values of 0.56 at 12 months, 0.55 at 36 months, and 0.62 at 60 months. Treatment complications included steroid-induced diabetes in four patients (1.4%) and AVN of the hip in one patient. Leucopenia occurred in 33 patients (11.5%), requiring dose reduction, while MMF was discontinued in two patients due to recurrent infections. Financial constraints led to the switch from MPA to azathioprine in three patients. By the end of 60 months, 35 patients (12.2%) were lost to follow-up, and 47 patients (27 males and 20 females, 16.4%) progressed to ESKD.

### Comparison of ESKD Progression

**Figure 2:**
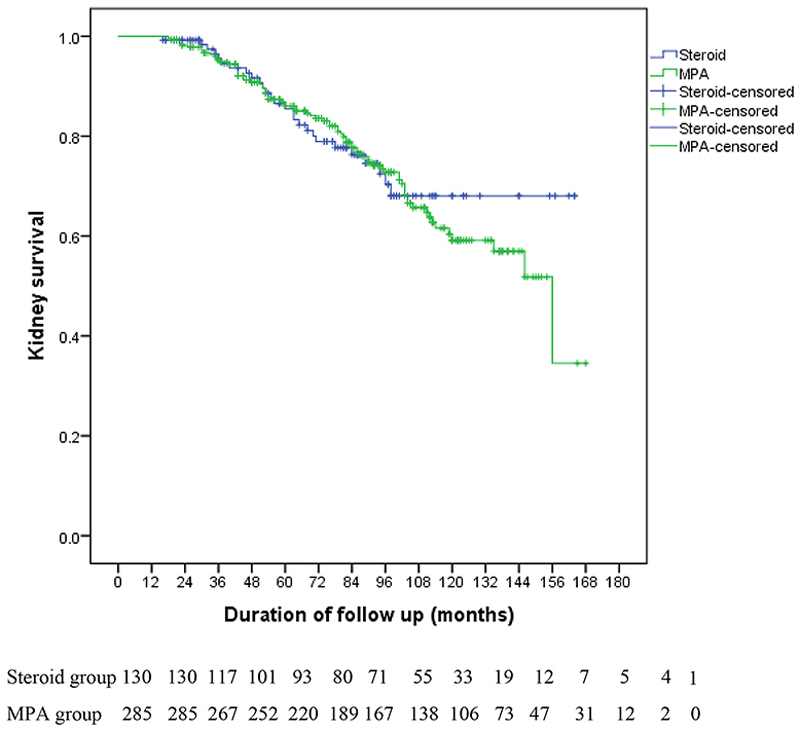
Survival analysis comparing Steroid and MPA groups. MPA: Mycophenolate.

At the end of the follow-up period, 18.4% of patients in the A+S group progressed to ESKD, compared to 16.4% in the A+S+M group. However, this difference was not statistically significant (P = 0.65). The estimated median time to progression to ESKD was 132.3 months (95% CI = 122–142.5) in the A+S group and 126.9 months (95% CI = 119.4–134.4) in the A+S+M group as depicted in Figure 2. The comparison of eGFR and proteinuria was between steroid and MPA groups was detailed in Table 2.

## Discussion

In this study, IgAN accounted for 16.7% of native kidney biopsies, a figure consistent with findings from the GRACE-IgANI^7^ study and an international survey on IgAN in Europe and North America.^8^ The mean age of patients was 36.6 ± 10.1 years, with a male-to-female ratio of 2:1, aligning with findings from European studies and the GRACE-IgANI study.^9–13^ Patients were treated with either non-immunosuppressive therapy (ACEi/ARB) or immunosuppressive treatment (steroids and MPA), depending on baseline eGFR and histological parameters. Steroid therapy, primarily recommended based on the TESTING and STOP IgAN studies,^14,15^ was supplemented by MPA, which was particularly suggested as a steroid-sparing agent for Chinese patients, according to the KDIGO 2022 guidelines.^16^ Although the target MPA level has not been defined for glomerular diseases, we adopted the MPA AUC range of 30-60 mg.h/L from kidney transplant protocols.^17,18^

Our results are consistent with prior studies investigating the role of MPA in IgAN. Chen *et al*.^19^ However, the difference in progression to ESKD between the steroid and MPA groups in our study did not reach statistical significance, echoing findings from other trials that reported mixed results regarding the benefits of steroid-sparing therapies.^20,21^ Notably, Frisch *et al*. found no benefit of MMF over renin-angiotensin system inhibitors in preventing ESKD,^20^ while Hou *et al*. reported similar remission rates with MMF and prednisone.^22^ The recent MAIN trial,^23^ compared mycophenolate with standard of care and concluded that 8.2% of patients in the Mycophenolate group progressed to ESKD, compared to 27.1% in the standard of care group. In the MAIN trial, the Mycophenolate group had a mean eGFR of 50.9 ± 18.2 ml/min/1.73m^2^ and Up/Uc of 2.1 ± 1.9, whereas the standard of care group had a mean eGFR of 49.3 ± 17.7 ml/min/1.73m^2^ and Up/Uc of 1.7 ± 1.3. A comparison of the present study with previous studies on Mycophenolate therapy in IgA nephropathy is detailed in Table 3.

The findings from this study highlight the potential role of MPA therapy as a disease-modifying treatment in IgAN. The addition of MPA to steroid therapy may offer a protective effect against progression to ESKD, as evidenced by a lower proportion of patients progressing to ESKD in the MPA group compared to the steroid-alone group, over a 60-month follow-up. This difference, was not statistically significant, but suggests a trend toward a more favorable long-term kidney outcome in the MPA group. Mesangial C3 deposition and the potential role of the complement system in disease progression is an area of interest, as it has been associated with adverse long-term outcomes in IgAN.^24–26^

Though it is one of the largest studies from India to assess the outcomes of MPA therapy in IgAN and compare them with steroid treatment, is has several limitations. First, its retrospective nature may introduce selection bias, as patients were treated according to clinical decision-making rather than randomized assignment. The sample sizes across the three groups were unequal, which may affect the generalizability of the findings. The incomplete documentation of side effects, particularly those related to drug use, limits the ability to fully assess the safety profile of the treatments. Moreover, the unavailability of MEST-C scoring for all patients reduces the precision of histological risk assessment. Despite these limitations, this study is the first large retrospective analysis from India to assess the outcomes of MPA therapy in IgAN and compare them to steroid treatment.

Future studies, particularly randomized controlled trials, are needed to validate the findings of this study. The development of a clear target MPA AUC for glomerular diseases would also help guide therapy in clinical practice. Investigating the role of the complement system in IgAN pathogenesis, particularly through the use of complement inhibitors like Iptacopan, could provide valuable insights into novel therapeutic strategies for IgAN. Prospective studies with larger sample sizes and long-term follow-ups are warranted to better evaluate the impact of MPA therapy on disease progression and long-term kidney outcomes in IgAN patients.

In conclusion, the lower percentage of patients undergoing CKD due to MPA therapy with steroids and ARBs, warrants an exploration through appropriately powered randomized controlled trials.

## Figures and Tables

**Table 1:** Baseline characteristics of study participants

Characteristics	ARB	ARB + Steroids	ARB + Steroids + MPA
No. of patients (n)	100	130	285
Age (yr)	34.22 ± 10.33	37.01±10.37	37.42±9.94
Male	60	70	61
Female	40	30	33
Hypertension n (%)	59	83	77.5
Known case of type 2 Diabetes n (%)	5 (5%)	7 (5.3%)	10 (3.5%)
Chronic HBV infection and on anti-viral treatment	-	1	2
Psoriasis Vulgaris	1	-	-
HIV infection and on ART	-	-	1
Low serum complement level n (%)	2/58 (3.4%)	5/74 (6.75%)	11/183 (6.01%)
Baseline eGFR (ml/min/1.73m^2^) at kidney biopsy	80.21 (50.98, 106.09)	48.73 (35.77, 75.84)	45.60 (32.00, 62.85)
Baseline Up/Uc at kidney biopsy	0.92 (0.47, 1.58)	1.59 (0.79, 2.54)	1.88 (0.97, 3.29)
Lost follow up at			
1 years	Nil	Nil	Nil
3 years	Nil	29 (22.3%)	33 (11.5%)
5 years	13 (13%)	50 (38.4%)	96 (33.6%)
No. of patients progressed to ESKD at			
12 months	01 (1%)	01 (0.76%)	04 (1.4%)
36 months	03 (3%)	10 (7.7%)	19 (6.6%)
60 months	08 (8%)	13 (10%)	24 (8.4%)
Total no. of patients progressed to ESKD	12 (12%)	24 (18.4%)	47 (16.4%)
MPA AUC (mg.h/L)	NA	NA	46.45+14.70
Steroid side effects	NA		
Diabetes		01	04
AVN Hip		Nil	01
MPA related side effects	NA	NA	
Leucopenia			33 (11.5%)
Infections			
Recurrent giardiasis			01
Disseminated TB			01

ARB: Angiotensin receptor blockers, ART: Anti-retroviral therapy, AUC: Area under curve, AVN: Avascular necrosis of Hip, eGFR: estimated glomerular filtration rate, ESKD: End Stage Kidney Disease, HBV: Hepatitis B virus infection, HIV: Human Immunodeficiency virus, IFTA: Interstitial fibrosis and tubular atrophy, MPA: Mycophenolic acid, TB: Tuberculosis, Up/Uc: Urine protein creatinine ratio. Age was expressed in Mean ± standard deviation, eGFR and Up/Uc– expressed in Median & Inter Quartile ranges (0.25, 0.75).

**Table 2:** Comparison of ARB+Steroid and ARB+Sterois+MPA groups

Variable	ARB + Steroid group	ARB + Steroid + MPA	p value
Age	37.04±10.43	37.41±9.94	0.73
eGFR (ml/min/1.73m^2^) at			
Baseline	48.73 (35.77, 75.84)	45.60 (32.00, 62.85)	0.01
12 months	57.74 (40.05, 86.58)	48.71 (37.07, 70.30)	0.02
36 months	50.00 (35.40, 83.84)	48.90 (33.95, 68)	0.13
60 months	49.21 (23.10, 82.10)	43.60 (26.05, 68.73)	0.42
Up/Uc (g/g) at			
Baseline	1.59 (0.79, 2.54)	1.88 (0.97, 3.29)	0.14
12 months	0.38 (0.15, 0.94)	0.56 (0.23, 1.10)	0.02
36 months	0.48 (0.15, 1.10)	0.55 (0.23, 1.00)	0.38
60 months	0.67 (0.21,1.17)	0.62 (0.26, 1.28)	0.56
No. of patients progressed to ESKD at 60 months	24	47	RR-0.96

Mann-Whitney test, Age expressed in mean + standard deviation, eGFR and Up/Uc– expressed in Median & Inter Quartile ranges (0.25, 0.75), Relative risk 0.96 and the chi-square statistic is 0.24, the p-value is 0.62. ARB: Angiotensin receptor blocker, MPA: mycopheolate acid, RR: Relative risk, Up/Uc: Urine protein creatinine ratio, eGFR: estimated glomerular filtration rate, ESKD: End stage kidney disease.

**Table 3:** Comparison of present study with previous studies on mycophenolate therapy in IgA Nephropathy

Parameter	Present study	Chen^19^ (2002)	Frisch^20^ (2005)	Hogg^21^ (2015)	Hou^22^ (2017)	MAIN Trial^23^
Place of study	India, 2024	China	America	Canada	China	China
Type of study	Retrospective study	RCT	RCT	RCT	RCT	RCT
Duration	2010-2017	2002	2005	2015	2017	2013-2015
No. of patients included	515	62	32	44	174	85
Comparing groups	MMF/Prednisolone	MMF/Prednisolone	MMF/RASi	MMF/Placebo	MMF/Prednisolone	MMF/Standard of care alone
Sample size in each group	130/285	31/31	17/15	22/22	86/88	85
Follow up	60 months	18 months	24 months	6 months	6 months	60 months
Outcomes assessed	eGFR/ proteinuria/ESKD	Clinical remission (Proteinuria)	Clinical remission/eGFR/ESKD	Complete remission (Up/Uc-0.2g/g)	Complete remission, ESKD	Doubling of creatinine, ESKD
Outcomes	Progressed to ESKD in MMF group - 16.4% Progressed to ESKD in steroid group - 18.4%	Remission rate MMF-44.4% Remission rate in steroid group - 19.1%	In both groups, all patients reached ESRD.	MMF did not reduce proteinuria significantly	At 12 months, complete remission rates were 48% and 53% in the MMF and prednisone groups, respectively (p-0.06)	Progressed to ESKD in MMF group - 8.2% Progressed to ESKD in other group 27.1%

RCT: Randomized controlled trial, MMF: Mycophenolate mofetil, ESKD: End stage kidney disease, RASi: Renin angiotensin aldosterone system inhibitors, Up/Uc: Urine protein creatinine ratio, eGFR: Estimated glomerular filtration rate.
